# Supplementary material for: Ankrd1 Promotes Lamellipodia Formation and Cell Motility via Interaction with Talin-1 in Clear Cell Renal Cell Carcinoma
Source: Int J Mol Sci. 2025 Apr 29;26(9):4232. doi: 10.3390/ijms26094232 (PMC12072362; doi:10.3390/ijms26094232)

Supplementary Table S1 Sequences of RT-qPCR primers

Primer	Sequence
Ankrd1_Forward	5′-TGATGCGGTGAGACTGAACC-3′
Ankrd1_Reverse	5′-CGTCTTCCCAGCACAGTTCTT-3′
GAPDH_Forward	5′-GCACCGTCAAGGCTGAGAAC-3′
GAPDH_Reverse	5′-TGGTGAAGACGCCAGTGGA-3′

Supplementary Table S2 Targeting sequences of Ankrd1 siRNA

siRNA	Sequence
siAnkrd1#1 (hs.Ri.ANKRD1.13.1)	5′-GAACCUGAAAUCAUUACGGAACCTG -3′
siAnkrd1#2 (hs.Ri.ANKRD1.13.2)	5′-CGACAGACUCUUAAUCAGUAAAUGT -3′
siAnkrd1#3 (hs.Ri.ANKRD1.13.6)	5′-UUUCUAUUUAUCAACGUGUUGUGAA-3′

Supplementary Table S3 Primary antibodies

Chemical	Dilution	Manufacturer	Catalog no.	RRID
Immunoblotting				
Ankrd1	1:1000	Proteintech	11427-1-AP	AB_2227402
Talin-1	1:1000	Abcam	ab71333	AB_2204002
YAP	1:1000	Cell Signaling Technology	14074	AB_2650491
pYAP^{s127}	1:1000	Cell Signaling Technology	13008	AB_2650553
ERK5	1:1000	Cell Signaling Technology	12950	AB_2798068
pERK5$^{T218/Y220}$	1:1000	Cell Signaling Technology	3371	AB_2140424
Bcl-2	1:1000	Cell Signaling Technology	15071	AB_2744528
Mcl-1	1:1000	Cell Signaling Technology	39224	AB_2799149
Bcl-xL	1:1000	Cell Signaling Technology	2764	AB_2228008
PARP	1:500	BD Transduction Laboratories	551025	AB_394009
Caspase3	1:1000	Cell Signaling Technology	14220	AB_2798429
E-cadherin	1:1000	BD Transduction Laboratories	610181	AB_397580
N-cadherin	1:1000	BD Transduction Laboratories	610920	AB_2077527
Vimentin	1:3000	BD Transduction Laboratories	550513	AB_393716
Src	1:1000	Cell Signaling Technology	2109	AB_2106059

pSrc^{Y416}	1:1000	Cell Signaling Technology	59548	AB_2936373
FAK	1:500	BD Transduction Laboratories	610087	AB_397494
pFAK^{Y397}	1:1000	BD Transduction Laboratories	611806	AB_399286
Flag	1:1000	FUJIFILM Wako Pure Chemical Corporation	018-22381	AB_10659453
α-Tubulin	1:1000	FUJIFILM Wako Pure Chemical Corporation	017-25031	
Immunofluorescence, PLA				
Ankrd1	1:100	Proteintech	11427-1-AP	AB_2227402
Cortactin	1:1600	Abcam	Ab81208	AB_1640383
Talin-1	1:100	Abcam	ab157808	
Immunohistochemistry				
Ankrd1	1:50	GeneTex	GTX115923	AB_10622683

Supplementary Figure S1

Kaplan–Meier survival analysis based on *Ankrd1* expression for a cohort of patients with ccRCC from TCGA database.

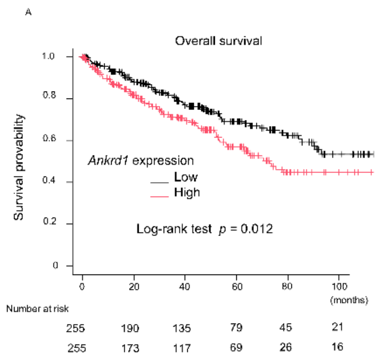

Supplementary Figure S2

Cell viability of 786-O and 769-P cells treated with increasing doses of XMD8-92 and XMD17-109 measured using the MTS assay.

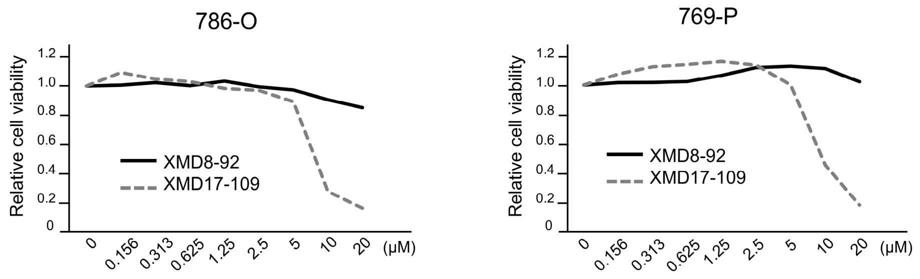

Supplement: Supplementary file 1 [file ijms-26-04232-s001.zip › ijms-3579838-supplementary.pdf]
